# Exploring the Nutritional Composition and Bioactive Compounds in Different Cocoa Powders

**DOI:** 10.3390/antiox12030716

**Published:** 2023-03-14

**Authors:** María del Carmen Razola-Díaz, María José Aznar-Ramos, Vito Verardo, Sonia Melgar-Locatelli, Estela Castilla-Ortega, Celia Rodríguez-Pérez

**Affiliations:** 1 Department of Nutrition and Food Science, Campus of Cartuja, University of Granada, 18011 Granada, Spain; 2 Biomedical Research Centre, Institute of Nutrition and Food Technology ‘José Mataix’, University of Granada, Avda del Conocimiento sn., 18100 Armilla, Spain; 3 Instituto de Investigación Biomédica de Málaga—IBIMA, 29071 Málaga, Spain; 4 Departamento de Psicobiología y Metodología de las Ciencias del Comportamiento, Facultad de Psicología, Universidad de Málaga, 29071 Málaga, Spain; 5 Department of Nutrition and Food Science, Campus of Melilla, University of Granada, C/Santander, 52005 Melilla, Spain; 6 Instituto de Investigación Biosanitaria ibs.GRANADA, 18012 Granada, Spain

**Keywords:** cocoa powder, phenolic compounds, methylxanthines, procyanidins, antioxidant activity

## Abstract

Cocoa, the main derivative of the seeds of *Theobroma cacao* L., has been recognized to have several effects on human health including antioxidant and neuro- and cardio-protective effects, among others. These effects have been attributed mainly to its bioactive compounds. In this context, the aim of this work is to evaluate the nutritional composition, bioactive compounds (i.e., phenolic compounds, procyanidins and methylxanthines) and the antioxidant activity of seven different cocoas (alkalized and non-alkalized) from different origins (Peru, Venezuela, Ivory Coast, Dominican Republic, and West Africa). It represents the first stage of a larger project aiming to find high polyphenol cocoa-based nutritional strategies and related biomarkers that may potentiate brain plasticity and cognitive function. Cocoa powders were extracted by ultrasound-assisted technology, and the total phenolic content (TPC) was measured by Folin–Ciocalteu. Methylxanthines (caffeine and theobromine) and procyanidin contents were determined by HPLC-FLD-DAD, and the antioxidant activity was assessed through DPPH, ABTS and FRAP assays. Non-alkalized cocoas showed higher phenolic and procyanidin contents and higher antioxidant activity compared to the alkalized ones. A strongly significant (*p* < 0.05) positive correlation between the antioxidant activity and the TPC, especially with the total procyanidin content, but not with methylxanthines was found. In conclusion, the non-alkalized cocoas, especially the one from Peru, were the best candidates in terms of bioactive compounds. The cocoa from Peru had a TPC of 57.4 ± 14.4 mg of gallic acid equivalent/g d.w., 28,575.06 ± 62.37 µg of catechin equivalents/g d.w., and 39.15 ± 2.12 mg/g of methylxanthines. Further studies should be undertaken to evaluate its effect on brain plasticity and cognitive function.

## 1. Introduction

Worldwide cocoa production has increased nearly 18% in the last ten years [1]. The global trends towards health and wellness, low-sugar, vegan, and allergen-free cocoa products have grown steadily. Premiumization, e.g., single origin or natural cocoa, is expected to be a major driver in the cocoa sector. According to recent data, the main cocoa producers are Cote d’Ivoire, Ghana, Brazil, Cameroon, Dominican Republic, Ecuador, Mexico, Nigeria, and Peru [2]. However, in terms of consumption, Europe is the main consumer, reaching 1.8 million tons in 2016 [3], with the UK (47 K tons), Germany (41 K tons) and Spain (35 K tons) being the main consumer countries in 2018 and representing 41% of the total cocoa consumption in Europe [4]. Cocoa is derived from the seeds of *Theobroma cacao* L., an evergreen tree typical of tropical regions. It contains numerous phytochemicals, with polyphenols representing the largest groups of compounds inside the seed; these have been reported to have several biological properties, such as antioxidant, antiapoptotic, anti-inflammatory and anti-cancer [5]. Moreover, cocoa’s effects have been investigated in different health conditions, including heart diseases, dyspepsia, nervous system diseases, blood circulation problems, and many others [5]. The main flavonoids contained in cocoa are procyanidins (flavan-3-ols). Concretely, these compounds are oligomers and polymers of catechin and epicatechin with different degrees of polymerization (DP) [6]. These compounds also have beneficial effects on the brain. They stimulate brain perfusion and allow angiogenesis, neurogenesis and changes in neuron morphology that favor learning and memory. Moreover, flavonoids preserve cognitive abilities and, therefore, they have been highlighted as compounds for reducing the risk of developing Alzheimer’s disease and stroke in humans [7]. In addition, the cocoa seed contains other phenolic compound such as flavonols (quercetin, isoquercetin), flavones (luteolin, apigenin), flavanones (naringenin), anthocyanins and phenolic acids. These compounds are highly related to antioxidant activity [8]. Other interesting compounds present in cocoa are methylxanthines. These alkaloids are non-selective adenosine receptor antagonists and competitive non-selective phosphodiesterase inhibitors [9]. Theobromine is the main one found in cocoa seeds, while caffeine and theophylline are found to a lesser degree [8]. These compounds have been reported to have physiological and psychological effects on humans such as neuroprotection, bronchodilation, diuresis, gastric secretion stimulation and metabolic effects, among others [8].

However, the highly variable content of phenolic compounds in cocoa and cocoa-derived products should be noted. In this regard, the highest content of polyphenols and methylxanthines is found in pure cocoa powder, followed by baking chocolate and dark chocolate, while the lowest polyphenol content is found in so-called white chocolate, which is made from the cocoa butter and barely contains methylxanthines [10,11,12]. Other factors such as the genotype of the cocoa plant—Forastero/Amazónico, Criollo or Trinitario—the region, method of cultivation and the manufacturing processes influence cocoa components significantly [13,14,15]. Moreover, phenolic compounds’ and methylxanthines’ composition could change due to the different cocoas origins and post-harvest processes [16]. Cocoa manufacturing involves fermentation, drying, roasting and Dutch process or alkalization stages. Alkalization is a widely used treatment to produce cocoa powder because it increases its solubility, adjusts its flavor and color, and reduces its astringency, bitterness, and acidic notes. This treatment consists of mixing cocoa with an alkali solution with a determinate temperature and pressure. Thus, depending on the pH, it can be obtained dark natural (pH 5 to 6), light (pH 6 to 7.2), medium (pH 7.2 to 7.6), or strong (pH > 7.6) alkalized cocoas. Alkalization could also produce undesirable nutritional and functional changes. In addition to affecting macronutrients, it can reduce the content of phenolic compounds and methylxanthines [17]. The reduction in these phenolic compounds could be explained by an increase in polyphenol oxidase activity and the oxidation and interaction of polyphenols with polysaccharides, proteins, other polyphenols, Maillard products, and pyrazines and their precursors. The methylxanthines’ reduction could be due to their interaction with alkali agents and their conversion into salts [8,17].

In the context of this research, evidence supports that cocoa consumption can potentiate cognitive function, but the actions of cocoa on the nervous system have been scarcely investigated. Cocoa polyphenols exert fast vasodilatory actions that increase brain perfusion and activate intracellular pathways related to brain plasticity, enhancing the synthesis of neurotrophic factors which can ultimately promote the birth of new neurons in specific regions of the adult brain. In this regard, adult hippocampal neurogenesis (AHN) is a form of neuroplasticity associated with improved learning and memory, emotional regulation, and protection from psychiatric and neurodegenerative diseases [18,19,20]. Nevertheless, the actions of cocoa on AHN are still unknown. For this reason and considering the highly variable phenolic composition of cocoa and cocoa-derived products, the study of cocoa powder’s composition to better understand its health effects is crucial. Thus, the aim of this work was to evaluate the nutritional labelling, phenolic compounds, procyanidins, methylxanthines and antioxidant activity of seven cocoas (alkalized and non-alkalized) from different origins and available on the Spanish market, for selecting the best one in term of bioactive compounds for future neurological in vivo studies.

## 2. Materials and Methods

### 2.1. Reagents

Gallic acid, Trolox, 2,2-diphenyl-1-picryl-hydrazyl-hydrate (DPPH), 2,2′-azino-bis (3-ethylbenzothiazoline-6-sulfonic acid (ABTS) and ferric reducing antioxidant power (FRAP) reagents and the pure standards catechin, caffeine and theobromine were acquired from Sigma-Aldrich (St. Louis, MO, USA), while Na_2_CO_3_ was purchased from BDH AnalaR (Poole, UK). Ultrapure water was obtained from a Milli-Q system (Millipore, Bedford, MA, USA). Lastly, HPLC-grade water, Folin–Ciocalteu reagent, and other reagents were acquired from Merck KGaA (Darmstadt, Germany).

### 2.2. Samples

Commercially available cocoa powder samples were purchased from Spanish local and specialized cocoa markets. Cocoa powders were far from the best before date of the labelling and were opened for the first time for the analyses. The list of the cocoa powders, indicating their origins and if alkalized or not, is shown in Table 1. The specific nutritional composition of each cocoa powder according to its labelling is shown in Table S1.

### 2.3. Ultrasound Bath Extraction

The extraction of bioactive compounds from cocoa powders was carried out by ultrasound technology, according to previous research, with slight modifications [21,22]. Firstly, the samples were defatted by adding 10 mL of hexane to 1 g of cocoa powder, vortexed 1 min, sonicated in ultrasound bath (Bandelin, Sonorex, RK52, Berlin, Germany) for 5 min, centrifugated at 9000 rpm 5 min and evaporated under nitrogen. This procedure was repeated twice. Then, the extraction was performed by adding 5 mL of a mixture of acetone/water/acetic acid 70/29.5/0.5; this was vortexed for 2 min, sonicated in an ultrasound bath (Bandelin, Sonorex, RK52, Berlin, Germany) which worked at a frequency of 35 kHz for 5 min, and centrifugated at 9000 rpm for 5 min. The extracting procedure was repeated twice, and the supernatants were collected. These extracts were filtered with regenerated cellulose filters 0.2 µm (Millipore, Bedford, MA, USA) and stored at −18 °C until the analyses.

### 2.4. Determination of Total Phenolic Content by Folin–Ciocalteu

The Folin–Ciocalteu spectrophotometric method was used to determine the total phenolic content (TPC) in cocoa powder samples [23]. Briefly, 100 µL of extract was added of 500 µL of the Folin–Ciocalteu reagent. Then, we added 6 mL of bi-distilled water, and the flask was kept in agitation for a minute. After that, 2 mL of 15% (*w*/*v*) Na_2_CO_3_ was added, and then the flask was filled up to 10 mL with bi-distilled water. Then, all the flasks were kept in a dark environment for 2 h, and the measurements were carried out at 750 nm and 25 °C with a UV-visible spectrophotometer (Spectrophotometer 300 Array, UV-Vis, single beam, Shimadzu, Duisburg, Germany). Gallic acid was used for the calibration curve from 1 to 1000 µg/g. The analyses were performed in triplicate, and the results are expressed as mg gallic acid equivalents (GAE)/g dry weight (d.w.).

### 2.5. Determination of Flavan-3-Ols by HPLC-FLD

The procyanidin content was determined in the cocoa samples by the methodology previously reported by López-Cobo et al. [24]. All the analyses were carried out at 35 °C. The identification of flavan-3-ols was carried out according to the previous HPLC-MS-ESI-TOF analyses and considering the elution of the compounds, because they elute depending on their degree of polymerization; they first elute the monomers and then the different oligomers [25]. To quantify flavan-3-ols, catechin was used as the standard at six concentration levels from 10 to 650 µg/g. In addition, application of the correction factors suggested by Robbins et al. was carried out [25]. Analyses were performed in triplicate and the results are expressed as mg catechin equivalents (CE)/g d.w.

### 2.6. Determination of Methylxanthynes by HPLC-DAD

The determination of caffeine and theobromine was carried out following the procedure described previously by Alañon et al. [26]. An Agilent 1200 Series (Agilent Technologies, Palo Alto, CA, USA), equipped with a quaternary pump delivery system, a degasser, an autosampler and a photodiode array detector (DAD) set up at 264 nm were used for the analyses. An Aagilent Zorbax Eclipse XDB-C18 column 5 µm, 150 × 4.6 mm ID (Agilent Technologies, Palo Alto, CA, USA) was used. Mobile phase A, B, C and D consisted of water (A), 200 mM sodium acetate/methanol 84/16 pH 4.4 (B), methanol (C) and acetonitrile (D). The gradient elution was: 25% B at 0 min, 25% B and 75% C for 3 min, 25% B and 50% D for 10 min and 25% B for 25–40 min. The injection volume was 100 µL, and the flow rate was 1 mL/min. Standard curves of caffeine and theobromine were performed at six concentration levels from 40 to 1250 µg/g for the quantification. The results are expressed as mg/g d.w.

### 2.7. Determination of Antioxidant Activity: DPPH, ABTS and FRAP Assays

The antioxidant activity of the cocoa powders was determined by DPPH, ABTS and FRAP methods, as described in previous research [27,28,29,30]. In all the assays, the calibration curve was made of the standard Trolox, and the results were expressed in mg of Trolox equivalents (TE)/g d.w. Analyses were performed in triplicate and the measurements were carried out using an UV–visible spectrophotometer (Spectrophotometer 300 Array, UV–Vis, single beam, Shimadzu, Duisburg, Germany).

### 2.8. Statistical Analysis

A one-way ANOVA (Tukey’s test) and Pearson’s correlation analyses were performed using the Statistica 7.0 package (StatSoft, Tulsa, OK, USA). Additionally, a correlation heatmap and principal component analysis (PCA) were performed by MetaboAnalyst. The significance level was selected as a 95% confidence interval (*p* < 0.05) for all the analyses. However, since the dataset is small, the significance level was extended up to *p* < 0.1 (90% confidence interval) in some cases; this is in agreement with the studies performed by other authors [31,32,33,34,35].

## 3. Results and Discussion

### 3.1. Nutritional Evaluation of the Cocoas

Table 2 summarizes the nutritional value collected from the labelling of cocoa powder samples. The energetic cocoa value ranges from 1249.8 to 1634.4 kJ/100 g. In the study of Jayeola and Oluwadun [36], the energetic value of the different cocoa powders from Nigeria was higher than that of our samples. The range included between 1523.0 and 2104.6 kJ/100 g of product. The energy input of cocoas could be attributed mainly to the fats present in it (r = 0.8019, *p* < 0.05), carbohydrates and fats being the main elements influencing total energy.

Carbohydrate values ranged from 9 to 28 g/100 g of product, with sugars being in a small proportion, i.e., less than 2 g/100 g cocoa in all the studied samples. The content of carbohydrates in cocoa and cocoa products is highly variable. In fact, different values of carbohydrates have been reported by other authors, i.e., about 50–60 g carbohydrates/100 g cocoa [36,37,38]. A decrease in sugars without changes in total carbohydrates was previously reported in alkalized cocoa products depending on the alkalization conditions [8]. However, no significant differences between the sugar content in the studied alkalized and non-alkalized cocoas were found. According to some authors [39], the sugar content and distribution in cocoas change during the processing. Initially, the characteristic sugars in cocoa beans are fructose and sucrose, but after the fermentation process, it is possible to also find glucose. Moreover, cocoa contains raffinose, stachyose and verbascose in smaller quantities. Finally, after roasting, the main sugars are glucose, galactose and arabinose [39]. It should be noted that many commercialized cocoa powders have added sugars [40]. In this regard, according to the Spanish users and consumer organization (OCU), the sugar content of soluble commercial cocoa products is too high, and it could represent 65% of the product [41]. When discussing dietary sugars (also called total sugars), all mono- and disaccharides that are present in the food, naturally or added, are considered [42]. Their intake could be associated with an increase in glycemic index, hyperglycemia, and type 2 diabetes mellitus (T2DM). Therefore, a high intake of dietary sugars could have undesirable health effects. Nevertheless, the studied samples have no added sugars, and those reported are naturally occurring in the cocoas, making them a healthier alternative to added sugar cocoa powders.

Cocoa powders can be classified according to their fat content. In European legislation, the product obtained by converting into powder cocoa beans which have been cleaned, shelled, and roasted, and which contains more than 20% cocoa butter and not more than 9% water, is called “cocoa powder”, whereas “fat-reduced cocoa powder” contains less than 20% cocoa butter [43]. Accordingly, cocoas 1, 2 and 5 were classified as “cocoa powder” (with fat content from 21 to 23 g/100 g), while cocoas 3, 4, 6 and 7 were classified as “fat-reduced cocoa powder” (with fat content ranging from 11 to 12 g/100 g) (see Table S1 for detailed information about fat composition). Those values agree with the ones previously published [17,21,22,23]. In general, fat-reduced cocoa powders showed a major carbohydrates content. Despite this, some authors have reported lower fat values in alkalized cocoa powders (4.93 g/100 g) [44], probably due to the hydrolysis and saponification of triglycerides [8]; in this study, this effect was not observed.

The content of proteins was ≈20 g/100 g of cocoa, except for cocoa 3 (non-alkalized) whose content was 28 g/100 g of cocoa. Petit et al. [45] reported protein values between 18.1 to 24.4 g/100 g of cocoa, with this value being higher in samples with lower fat content. In the study of Adeyeye [44], a content of 10.9 g/100 g of alkalized cocoa powder was observed. That diminution of protein content could be due to oxidative destruction by deamination [8]. However, this reduction was not observed in the cocoa powder samples evaluated in this study. This fact could be due to the employed alkalization process. The alkali concentration and thus the pH will lead to the formation of brown compounds in the alkalized cocoa powders through partial protein deamination [46]. Therefore, the employed alkalization process could influence the protein content in cocoa powders.

Finally, the salt content is very close to 0 g/100 g of product; thus, cocoa powder was not characterized as a product with high salt content.

### 3.2. Total Phenolic Content of Cocoas

**Figure 1:**
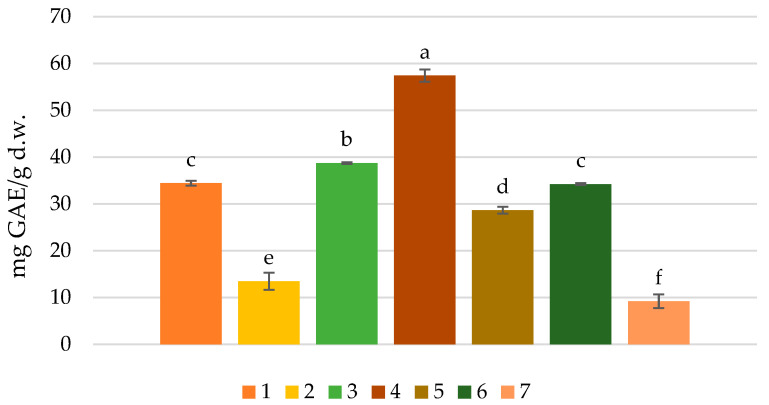
Total phenolic content of the analyzed cocoas as analyzed by Folin–Ciocalteu. Different letters indicate significant differences. Numbers 1–7 correspond to the cocoa samples.

The total phenolic content (TPC) of the seven cocoa extracts was measured by the Folin–Ciocalteu method. The obtained results (expressed as mg gallic acid equivalent (GAE)/g d.w.) are shown in Figure 1.

As can be seen, the TPC ranged from 9.2 to 57.4 mg GAE/g d.w. Cocoa 7 (West Africa) had the lowest TPC, followed by cocoa 2 (Ivory Coast). Precisely, those two cocoas were submitted to an alkalization process, also known as Dutching, which is usually employed to reduce the cocoa’s bitterness and to darken chocolate with the consequent loss of these compounds. This effect was previously reported by Li et al. [17]. They discovered that the content of total polyphenols decreased with the increasing degree of alkalization in cocoa powders. Additionally, they found that the polyphenols in cocoa interact with aroma precursors as well as aroma compounds, and with hydrophobic amino acids during the alkalization process. This can be attributed to the high temperatures and the exposure of the cocoa powders to oxygen during the alkalization process [17]. Within the non-alkalized cocoas, it is also possible to observe significant differences in TPC that could be influenced by the geographical origin, as previously reported [47]. In this regard, the cocoa from Peru (cocoa 4) presented the highest value of TPC (57.4 mg GAE/g d.w). That result is in line with that reported by Ramos-Escudero et al. for Peruvian cocoa beans (21.88–33.39 mg GAE/g) [48] and that of Grassia et al. for Peruvian cocoa (11.2 mg catechin equivalents/g) [49]. Additionally, the TPC values for non-alkalized cocoas were in line with those reported by Miller et al. [12] (from 45.3 to 60.2 mg GAE/g), Samaniego et al. [32] (from 33.6–71.7 mg GAE/g) for different cocoa powders, and Kobori et al. [50] for a cocoa powder from Ghana (62.3 mg epicatechin equivalents/g). Analyzed cocoa powder 1 from Venezuela had a TPC of 34.4 mg GAE/g d.w; this is in the same range of magnitude as the results reported in cocoas from other regions of south America, with values that ranged from 44–202 mg GAE/g in cocoa beans from Colombia [33,51,52] and from 33.6–71.7 mg GAE/g in cocoa beans from Equator [32].

**Figure 2:**
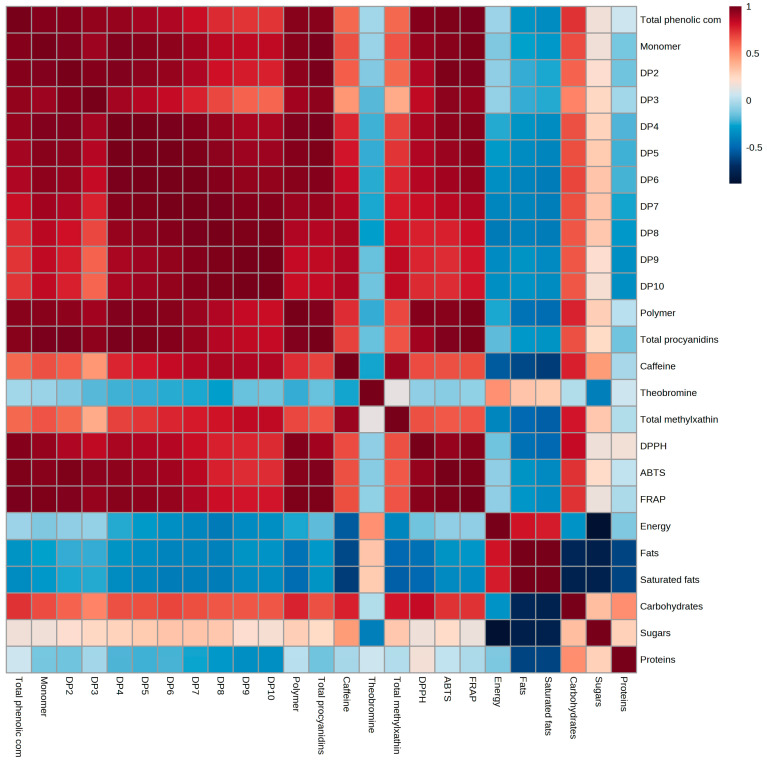
Pearson correlations heatmap of the cocoa powders’ analyses and nutritional facts. DP, degree of polymerization.

Figure 2 shows a Pearson correlations heatmap of the cocoa powders’ analyses and nutritional facts. A significant (*p* < 0.1) positive correlation between TPC and the carbohydrate content (r = 0.7306) was found. In fact, cocoas 3 and 4, which were which outlined higher polyphenol content, were those that had more carbohydrates. Those correlations can be attributed to the fact that a high carbohydrate content with high phenolic content can promote melanoidins’ formation when the product is subjected to heating in the roasting step [53]. Besides, some studies [54] have reported that melanoidins are carbohydrate–phenol structural compounds, thus explaining this positive correlation between carbohydrates and polyphenols in these cocoa powder samples. The same trend has been appreciated previously by other authors in cocoa powders, correlating the polyphenol content and the non-fat cocoa solids [6,55,56].

In addition, a PCA is shown in Figure S1, wherein component 1 and 2 means 99.5 and 0.3%, respectively. Here, we can clearly observe the differences between alkalized and non-alkalized cocoa powders.

### 3.3. Procyanidin Content of Cocoas

**Figure 3:**
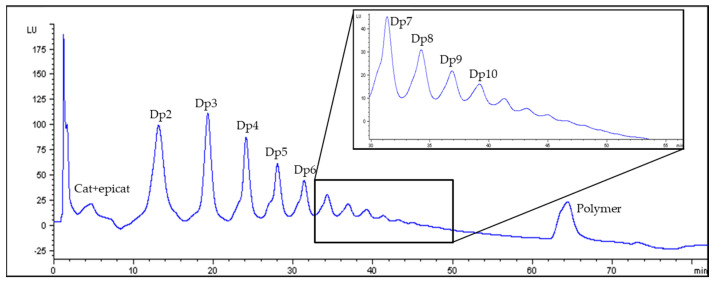
Procyanidin profile of one of the cocoas as analyzed by HPLC-FLD.

Table 3 summarizes the total procyanidin content of the cocoa powders, according to their degree of polymerization (DP) as analyzed by HPLC-FLD. In addition, a representative chromatogram of the procyanidin distribution is depicted in Figure 3.

The total flavan-3-ols content ranged between 3299 and 28,575 µg CE/g d.w. in the analyzed cocoas. The alkalized cocoas (cocoa 2 and 7) presented the lowest content in procyanidins, while the non-alkalized cocoas, especially those from Peru (cocoa 4), showed the highest content. Regarding the procyanidin profile, the major flavan-3-ols were those with low DP. Cat + epicat accounted for between 27–61% of the total content, followed by procyanidin dimer, which accounted for nearly 30% of the total flavan-3-ols in all cocoa samples. Contrarily, polymers with a DP higher than 10 ranged between 3 and 8.5% of the total procyanidin content. González-Barrio et al. reported values of 2.7 mg/g of cat + epicat, them being 16% of the total procyanidins in conventional cocoa powder, as quantified by HPLC-FLD. However, the same authors found around 37% of polymers had a polymerization degree higher than 10 [22]. Similar values of cat + epicat were found in the studied samples, with cocoa 4 (natural, from Peru) being the cocoa that presented the highest content. However, Ramos-Escudero et al. reported values up to 25.22 mg/g of cat + epicat in cocoa beans from Peru [48], and values of 10.9 and 11.5 mg/g were found in beans from Peru and Ghana, respectively [49]. Other studies reported values of 8 and 4 mg/g (monomer and dimer, respectively) in Colombian cocoa beans [52], and of 20 and 11 mg/g (monomer and dimer, respectively) in Equatorian cocoa beans [32], which are very similar values to those reported here for the natural Venezuelan cocoa powder (cocoa 1). In other parts of the cocoa bean, such as the shell, Botella-Martínez et al. obtained epicatechin and catechin as major flavonoids, with values that ranged from 4.56–6.33 and from 2.11–4.56 mg/g, respectively, in cocoa beans from Ghana [57]. Besides, in extracts from cocoa pods, some authors obtained values of 95.4 mg/g for monomers and 7.5 mg/g for dimers [58]. Those results agree with the data presented here, despite the differences in origin.

Interestingly, other authors reported a significant increase in catechin content with alkalization with temperature, time and the alkali NaOH process, but a significant reduction in the epicatechin content of Forastero cocoa with 10–12% fat [59,60]. Similar results, i.e., a 40% increase in catechin of, but a 23–66% reduction in epicatechin and proanthocyanidins, were reported by Stanley et al. when cocoa powder was alkalized with NaOH (final pH 8.0) at 92 °C for up to 1 h [61]. In the present study, although alkalized cocoas were those with the lowest absolute content of monomers and procyanidins, they had the same tendency regarding proportions. Thus, analyzed alkalized cocoa had a cat + epicat content ≥ 40%. In the case of cocoa 7 (alkalized from West Africa), an increment of 54% was found in the monomer content regarding the proportion and distribution. It would mean that the alkalization process probably destroys the linkage between catechin or epicatechin monomers in procyanidins with a higher degree if polymerization.

It has been previously reported that high-molecular-weight polymers of flavan-3-ols are poorly absorbed through the gastrointestinal tract. So, the efficiency of absorption of procyanidins decreased with the increasing degree of polymerization, and only small amounts of intact oligomers of procyanidins might be partially absorbed in the intestinal mucosa [62]. Therefore, compounds with low molecular weight, such as flavan-3-ol monomers and dimers, can achieve higher concentrations in the blood and reach the target organs in the body. According to this, natural cocoa from Peru (cocoa 4) could be the most bioactive cocoa due to its higher content in monomer and dimers compared to the other analyzed cocoas. A dose–response effect has also been described on plasma of cocoa epicatechin around 30–60 min after consumption, and a maximum plasma concentration has been observed 2–3 h after ingestion of flavan-3-ol-rich cocoa products [62]. Most of the bioactivities described for cocoa flavan-3-ols are attributed to their effect on the mitogen-activated protein kinase and phosphoinositide-3-kinase-protein kinase B/protein kinase B molecular signaling pathways. Cocoa procyanidins have been recognized as inhibitors of neuroinflammation; they are preventive against myofibroblasts’ transformation in cardias fibrosis and preventive/therapeutic in life-threatening diseases such as cancer and diabetes. Moreover, cocoa flavanols’ metabolites from catechin and epicatechin have been reported to improve glucose-stimulated insulin. Going further, cocoa epicatechin and its colonic metabolite 3,4-dihydroxyphenylacetic acid have been reported to protect the renal proximal tubular cell against high glucose-induced oxidative stress [63]. Additionally, procyanidin in cocoa powder has been reported to reduce LDL oxidative susceptibility and to have beneficial effects on plasma HDL-cholesterol concentrations in humans [64,65].

Moreover, a significant (*p* < 0.05) positive correlation was found between total procyanidin content and total phenolic content (r = 0.9643) (Figure 2). This makes sense because in fact, flavan-3-ols are the major polyphenols present in cocoa powders that form part of the total phenolic content determined by Folin–Ciocalteu, so they contribute the most to its total phenolic composition.

### 3.4. Methyxanthines Content of Cocoas

The caffeine and theobromine contents were analyzed by HPLC-DAD. The obtained results, including the theobromine/caffeine (T/C) ratio, are summarized in Table 4.

The total methylxanthine content ranged between 19.4 (natural cocoa from Dominican Republic) and 39.2 mg/g (natural cocoa from Peru), while caffeine and theobromine results ranged from 5.4 to 27.1 and from 8.87 to 15.1 mg/g, respectively. Natural cocoa from West Africa (cocoa 6) was the cocoa with the lowest theobromine content, and natural cocoa from Venezuela (cocoa 1) had the highest. Different trends were found for caffeine content. In this case, natural cocoa from the Dominican Republic (cocoa 5) was the one with the lowest caffeine content, while Peruvian natural cocoa (cocoa 4) had the highest content by some way. Comparing these results with those previously published, Alvarez et al. reported caffeine and theobromine values of 6.1 mg/g and 9.10 mg/g, respectively, with a T/C ratio of 1.5 in Venezuela Criollo cocoa beans [16]. In Equatorian cocoas, the theobromine and caffeine content ranged from 1.5 to 2.4 and from 0.2 to 0.4 mg/100 g, respectively [32]. Ramos-Escudero et al. reported values up to 12.95 mg/g of theobromine in Peruvian cocoa beans [48]. According to Grassia et al., Ghana’s cocoa beans had a lower caffeine content (0.2 mg/g) than Peruvian cocoa beans, but the highest theobromine concentration (10.4 mg/g) [49]. Other authors reported values of 2.9 and 21.5 mg/g of caffeine and theobromine, respectively, in non-alkalized cocoa beans from Ghana, with reductions after the alkalization process [66]. In cocoas from Peru, some authors reported ranges of 57.4–76.0 and 10.6–20.8 mg/g of theobromine and caffeine, respectively, with T/C ratios > 3 in all cases [67]. Similar results have been shown by other authors in cocoas from different regions of South America, with T/C ratios higher than 2 in all cases [33,51,52]. Overall, the present results are in line with those already published. Regarding the T/C ratio, cocoas 4 (natural from Peru), 6 (natural from West Africa) and 7 (alkalized from West Africa) had ratios < 1, while cocoas 1 (natural from Venezuela), 2 (alkalized from Ivory Coast) and 3 (natural from Ivory Coast) had ratio > 1. The only cocoa that presented a ratio higher than 2 was the one from the Dominican Republic (cocoa 5). As can be appreciated, the ratio seems to have origin dependency. Sioriki et al. analyzed alkalized and non-alkalized cocoa powders finding no statistical differences in the theobromine and caffeine content. They reported values of 7.4–8.3 mg/g in theobromine and 2.1–2.2 mg/g in caffeine [59]. In another study, they confirmed that no significant reductions or increments take place in cocoa during alkalization process due to temperature, time or alkali concentration [60]. In this regard, the results found in this study agree with those related to the total methylxanthines and caffeine content, but different results were obtained for theobromine. In this case, a reduction of 33% of theobromine was found from cocoa 6 to cocoa 7; these two are from the same market label, but the first one is alkalized and the second non-alkalized.

Some authors have found that cocoa methylxanthines mediate an increased plasma concentration of (−)-epicatechin metabolites, thus enhancing the effects of cocoa flavanols on cardiovascular function [68]. A moderately strong correlation was found between the total methylxanthine content and the total phenolic (r = 0.6096) and procyanidin (r = 0.6773) content. However, those correlations were insignificant.

### 3.5. Antioxidant Activity of Cocoas by DPPH, ABST and FRAP

**Figure 4:**
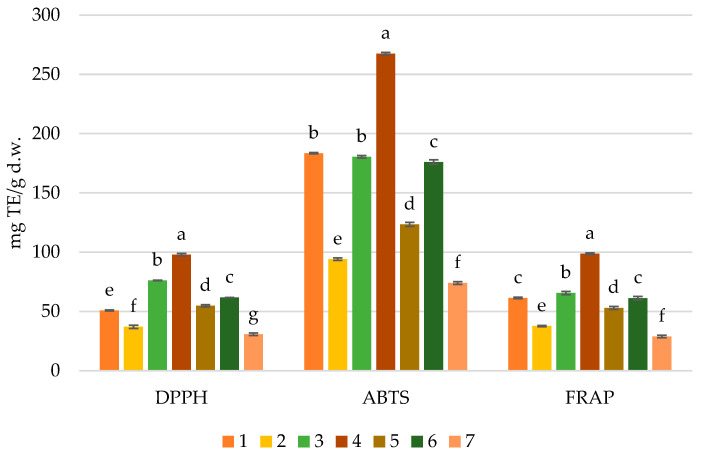
Antioxidant activity measured by DPPH, ABTS and FRAP assays of the analyzed cocoa powders. Different letters for each method indicate significant differences. Numbers 1–7 correspond to the cocoa samples.

The antioxidant activity of the cocoa powders was measured by three spectrophotometric methods, i.e., DPPH, ABTS and FRAP. The results are shown in Figure 4.

DPPH values ranged from 30.77 to 97.94 mg TE/g d.w. For ABTS, the values were from 73.97 to 267.43 mg TE/g d.w., and for FRAP assay from 28.88 to 98.74 mg TE/g d.w. As expected, and considering the abovementioned discussed data, alkalized cocoas (i.e., cocoa 2 and 7) had the lowest antioxidant activity, while cocoa 4 (natural, from Peru) presented the highest activity, according to the three different assays. Our results agree with those reported by Todorovic et al. [35]. They compared alkalized and non-alkalized cocoas in terms of antioxidant activity and reported ranges of 63.4–96.8, 56.9–65.7 and 87.1–113.8 mg TE/g d.w. for DPPH, ABTS and FRAP, respectively, in non-alkalized cocoas extracts, and lower ranges in the alkalized ones (i.e., 32.1–67.9, 28.2–57.0 and 27.6–77.4 mg TE/g d.w. by DPPH, ABTS and FRAP, respectively). Botella-Martínez et al. measured DPPH, ABTS and FRAP antioxidant assays in Ghanaian cocoa bean shells, with values of 2.35–5.53, 3.39–11.55, and 3.84–7.62 mg Trolox equivalents/g sample, respectively [57]. In Malaysian cocoa powder, some authors reported 47.2, 65.77, 85.8% inhibition by DPPH at concentrations of 5, 10 and 20 mg/mL, respectively [69]. The cocoa powder that had the highest percentage of inhibition for DPPH at 20 mg/mL was the one from Peru (cocoa 4), with 64% of inhibition. Besides, non-alkalized cocoa powders showed >60% inhibition, and the alkalized cocoa powders showed >50%, in all cases at 20 mg/mL.

The three methods showed significant (*p* < 0.05), strong, positive correlation between each other (DDPH vs. ABTS, r = 0.9284; DPPH vs. FRAP, r = 0.9630; ABTS vs. FRAP, r = 0.9842). Furthermore, they showed the highest significant (*p* < 0.05) positive correlation with the TPC, followed by the total procyanidin content (r > 0.9). Taking a significance level *p* of 0.1, a significant positive correlation was also observed between total methylxanthines, and the antioxidant activity measured by DPPH (0.6773) and FRAP (r = 0.6700); this is mainly attributable to the caffeine content. Additionally, a light negative correlation was found with the theobromine content. Previously, Kobori et al. reported that in cocoa powder with 80% reduced caffeine, the polyphenol content mas maintained 84%, and its antioxidant activity 85%, compared to the non-decaffeinated one [50]. So, in accordance with them and with the statistical data, it can be affirmed that the antioxidant activity of the cocoa powders is attributable to its phenolic and procyanidin content.

## 4. Conclusions

Non-alkalized (natural) and alkalized cocoas from Venezuela, the Ivory Coast, Peru, the Dominican Republic, and West Africa have been analyzed in terms of their energy and macronutrients reported in their labelling and their bioactive compounds’ composition. Differences in the nutritional composition of the studied cocoas have been observed. Their fat content allowed the cocoa to be classified as “cocoa powder” or “fat-reduced cocoa powder”. It has been found that fat-reduced cocoa powders have a higher carbohydrate content. Interestingly, no significant differences in carbohydrates, fats, and proteins between alkalized and natural cocoas have been found. In addition, the present results showed high differences in the total phenolic, procyanidins and methylxanthines contents depending on the cacao origin and treatment. In this regard, the alkalization process significantly reduced the concentration of those compounds, excepting the methylxanthine content. The natural Peruvian cocoa was the one that showed a significantly higher content of phenolic compounds, total procyanidins and total methylxanthines, and it presented the highest antioxidant activity measured by the three different assays. Meanwhile, alkalized cocoa from West Africa showed the lowest values except for total methylxanthines, with the natural cocoa from Dominical Republic having the lowest methylxanthines concentration. A significant and negative correlation between the fat content and the TPC was found. Contrarily, the TPC was positively associated with the procyanidin concentration. Finally, the antioxidant activity of the cocoa powders was directly related to its phenolic and procyanidin content, but not to the methylxanthines content. It should be highlighted that scarce information regarding the origin, bioactive compound composition or treatment is available on cocoa powder labels, thus, making difficult to compare the different options available in the market and to choose the most suitable to study its potential health effects. This fact, together with the lack of standardized analytical methods focused on measuring the aforementioned cocoa powder compositions, makes this type of research necessary prior to carry out in vivo and clinical studies.

## Figures and Tables

**Table 1:** List of the cocoa powders analyzed with their code name and origin.

Sample	Origin	Alkalized
1	Venezuela	N
2	Ivory Coast	A
3	Ivory Coast	N
4	Peru	N
5	Dominican Republic	N
6	West Africa	N
7	West Africa	A

N: Non-alkalized (natural); A: Alkalized.

**Table 2:** Summary of the nutritional labelling of the studied cocoa powders, per 100 g of product.

Per 100 g of Product	Energy (kJ)	Fat (g)	Saturated Fat (g)	Carbohydrates (g)	Sugars (g)	Protein (g)	Salt (g)
Average	1457.6	16.1	9.8	16.3	1.1	21.5	0.03
SD	158.4	5.84	3.60	7.99	0.73	3.26	0.02
Min.	1249.8	11.0	6.5	9.0	0.0	19.0	0.00
Max.	1634.4	23.0	14.0	28.0	2.0	28.0	0.06
CV (%)	10.9	36.2	36.6	49.0	67.9	15.2	80.9

SD: Standard deviation; CV: Coefficient of variation.

**Table 3:** Flavan-3-ol content of cocoa extracts analyzed by HPLC-FLD and expressed as average ± standard deviation.

	1 (µg CE/g d.w.)	2 (µg CE/g d.w.)	3 (µg CE/g d.w.)	4 (µg CE/g d.w.)	5 (µg CE/g d.w.)	6 (µg CE/g d.w.)	7 (µg CE/g d.w.)
Cat + Epicat	4536.90 ± 11.81 b	2238.23 ± 11.90 f	4288.26 ± 12.33 c	7816.56 ± 16.66 a	4132.57 ± 10.13 e	4201.90 ± 10.37 d	1999.86 ± 9.85 g
Dp2	5018.78 ± 12.39 b	1607.62 ± 10.41 f	3744.19 ± 9.41 d	7972.94 ± 17.33 a	3196.20 ± 8.22 e	4476.44 ± 11.02 c	928.93 ± 13.33 g
Dp3	2311.63 ± 12.86 c	589.02 ± 2.15 f	1731.81 ± 6.74 d	3141.32 ± 6.48 a	1538.89 ± 3.71 e	2462.54 ± 7.51 b	233.43 ± 2.63 g
Dp4	1191.32 ± 18.37 c	250.11 ± 2.26 f	902.74 ± 2.91 d	3268.16 ± 6.91 a	743.68 ± 2.19 e	1403.49 ± 3.29 b	28.94 ± 3.58 g
Dp5	612.22 ± 7.47 c	93.72 ± 1.82 f	447.36 ± 1.68 d	2018.99 ± 4.67 a	351.58 ± 1.23 e	796.08 ± 1.49 b	<LOQ
Dp6	228.95 ± 1.44 c	2.38 ± 0.76 f	180.24 ± 0.95 d	996.17 ± 2.10 a	113.11 ± 0.32 e	339.24 ± 0.99 b	<LOQ
Dp7	124.31 ± 0.69 c	<LOQ	79.71 ± 0.57 d	795.09 ± 2.06 a	43.24 ± 0.45 e	221.48 ± 1.25 b	<LOQ
Dp8	1.16 ± 1.52 c	<LOQ	<LOQ	330.58 ± 1.00 a	<LOQ	63.11 ± 0.42 b	<LOQ
Dp9	<LOQ	<LOQ	<LOQ	112.03 ± 0.65 a	<LOQ	1.78 ± 0.32 b	<LOQ
Dp10	<LOQ	<LOQ	<LOQ	28.94 ± 0.56	<LOQ	<LOQ	<LOD
Polymers	729.98 ± 3.99 d	252.95 ± 2.86 f	1023.22 ± 1.35 c	2094.28 ± 3.95 a	608.21 ± 1.38 e	1132.00 ± 2.73 b	105.18 ± 6.25 g
Sum of Procyanidins	14,756.84 ± 70.56 c	5035.55 ± 32.17 f	12,397.53 ± 35.95 d	28,575.06 ± 62.37 a	10,727.47 ± 27.62 e	15,101.75 ± 39.40 b	3298.89 ± 35.64 g

LOQ: Limit of quantification. CE: Catechin equivalents. Cat + Epicat: Catechin + epicatechin. Different letters (a–g) in the same line indicate significant differences.

**Table 4:** Methylxanthines content of cocoa extracts analyzed by HPLC-DAD and expressed as average ± standard deviation.

Sample	Caffeine (mg/g)	Theobromine (mg/g)	Total Methylxanthines (mg/g)	T/C Ratio
1	9.56 ± 0.21 ^c^	15.14 ± 0.38 ^a^	24.70 ± 0.59 ^b–d^	1.58
2	11.23 ± 0.16 ^b,c^	12.30 ± 1.87 ^a,b^	23.53 ± 1.89 ^b–d^	1.10
3	13.78 ± 0.60 ^b^	14.69 ± 3.41 ^a^	28.47 ± 3.61 ^b^	1.07
4	27.06 ± 1.34 ^a^	12.09 ± 2.06 ^a,b^	39.15 ± 2.12 ^a^	0.45
5	5.41 ± 0.22 ^d^	14.04 ± 0.76 ^a^	19.44 ± 0.87 ^d^	2.60
6	13.62 ± 0.06 ^b^	8.87 ± 0.44 ^b^	22.49 ± 0.49 ^c,d^	0.65
7	14.24 ± 0.57 ^b^	13.29 ± 0.08 ^a,b^	27.54 ± 2.93 ^b,c^	0.93

T/C: Theobromine/caffeine. Different letters (a–d) in the same column indicates significant differences (*p* < 0.05).

## Data Availability

Not applicable.

## Supplementary Materials

The following supporting information can be downloaded at: https://www.mdpi.com/article/10.3390/antiox12030716/s1, Table S1. Nutritional labelling of the studied cocoa powders per 100 g of product. Figure S1. Principal component analysis of the analyzed cocoas, according to its their nutritional labelling and the analyses performed.
