# Supplementary material for: Exploring the Nutritional Composition and Bioactive Compounds in Different Cocoa Powders
Source: Antioxidants (Basel). 2023 Mar 14;12(3):716. doi: 10.3390/antiox12030716 (PMC10045957; doi:10.3390/antiox12030716)
Supplement: Supplementary file 1 [file antioxidants-12-00716-s001.zip › antioxidants-2231869-supplementary.pdf]

Table S1. Nutritional labelling of the studied cocoa powders, per 100g of product.

Per 100 g of product	1	2	3	4	5	6	7
Energy (kJ)	1634.4	1605.1	1534.1	1337.6	1546.6	1295.8	1249.8
Fat (g)	23	23	11	12	21	11	12
Saturated fat (g)	14	14	6.5	7	13	7	7.4
Carbohydrates (g)	12	9	27	28	9	15	14.09
Sugars (g)	0.9	0.04	0.9	1.4	0.4	2	1.87
Protein (g)	20	19	28	19	19.4	23	21.96
Salt (g)	0.05	0.04	<0.1	0	0.04	0.06	0.025

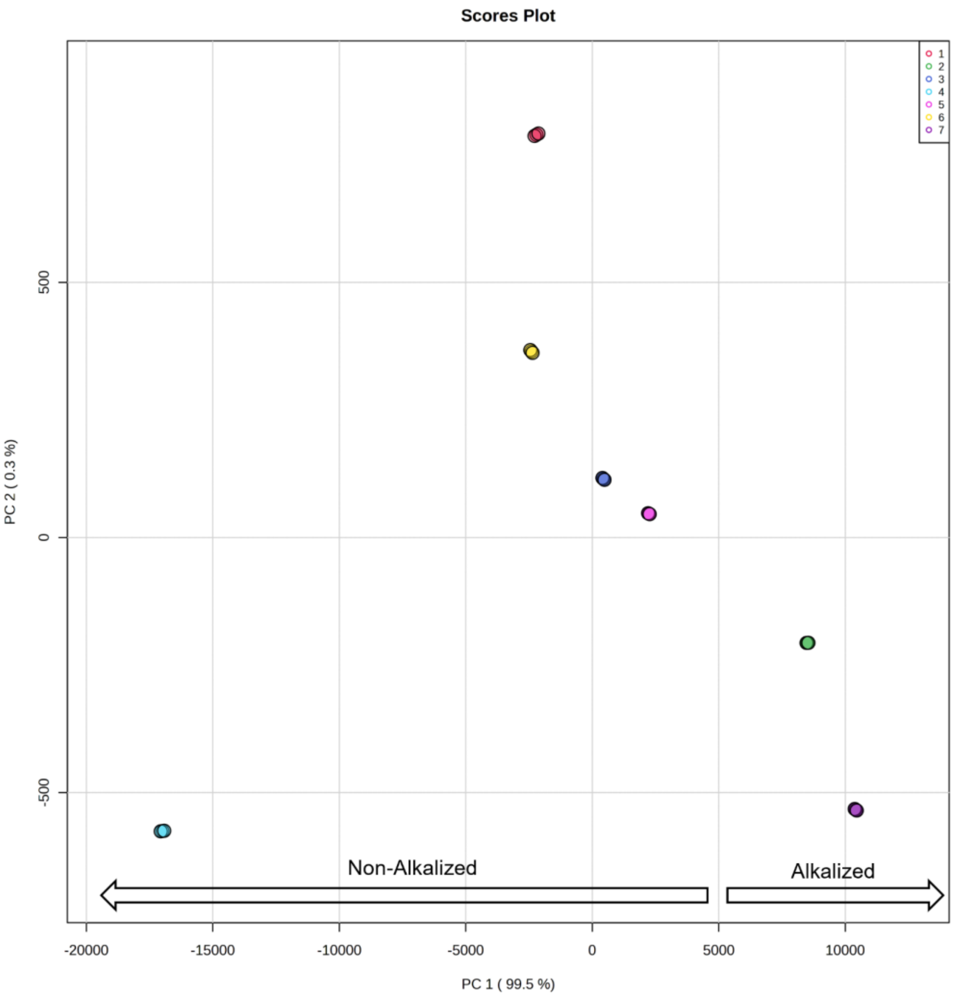

Figure S1. Principal component analysis of the analyzed cocoas, according to their nutritional labelling and the analyses performed.
